# Iron deposits in the chronically inflamed central nervous system and contributes to neurodegeneration

**DOI:** 10.1007/s00018-013-1509-8

**Published:** 2013-11-12

**Authors:** Hjalte Holm Andersen, Kasper Bendix Johnsen, Torben Moos

**Affiliations:** Laboratory for Neurobiology, Biomedicine Group, Department of Health Science and Technology, Aalborg University, Fr. Bajers Vej 3B, 1.216, 9220 Aalborg East, Denmark

**Keywords:** Blood–brain barrier, Cell death, Iron accumulation, Macrophage, Neurodegeneration, Nitric oxide, Phagocytosis

## Abstract

Neurodegenerative disorders are characterized by the presence of inflammation in areas with neuronal cell death and a regional increase in iron that exceeds what occurs during normal aging. The inflammatory process accompanying the neuronal degeneration involves glial cells of the central nervous system (CNS) and monocytes of the circulation that migrate into the CNS while transforming into phagocytic macrophages. This review outlines the possible mechanisms responsible for deposition of iron in neurodegenerative disorders with a main emphasis on how iron-containing monocytes may migrate into the CNS, transform into macrophages, and die out subsequently to their phagocytosis of damaged and dying neuronal cells. The dying macrophages may in turn release their iron, which enters the pool of labile iron to catalytically promote formation of free-radical-mediated stress and oxidative damage to adjacent cells, including neurons. Healthy neurons may also chronically acquire iron from the extracellular space as another principle mechanism for oxidative stress-mediated damage. Pharmacological handling of monocyte migration into the CNS combined with chelators that neutralize the effects of extracellular iron occurring due to the release from dying macrophages as well as intraneuronal chelation may denote good possibilities for reducing the deleterious consequences of iron deposition in the CNS.

## Introduction

### Neurodegenerative disorders are accompanied by inflammation and iron deposition

The most prevalent neurodegenerative disorders of the central nervous system (CNS) are characterized by their chronic affection of specific neuronal nuclei or regions, which leads to various clinical phenotypes (Table 1). Collectively, the loss of neurons in neurodegenerative disorders leads to a gradual loss of functional capacity with largely irreversible symptoms. The time course from the initiation of neuronal cell death to the appearance of clinical symptoms varies but generally decades span until a sufficient amount of neurons are affected, which opens therapeutic possibilities, the focus being to halt further neuronal loss [1].

**Table 1:** Neurodegenerative disorders with inflammation and accumulation of iron- and ferritin-containing macrophages

Disorder	Pathological features associated with neuronal degeneration	Affected CNS region accompanied by iron accumulation	References
Alzheimer’s disease	Intracellular deposition of neurofibrillary tangles containing hyperphosphorylated tau-protein and extracellular deposition of amyloid	Cerebral cortex, hippocampus	[138–143]
Parkinson’s disease	Aggregate-like structures formed by alpha-synuclein in affected dopaminergic neurons leading to formation of solid inclusion bodies known as Levy Bodies	Substantia nigra, striatum	[61, 144–152]
Huntington’s disease	Mutation in the gene encoding the huntingtin protein leading to accumulation of aggregates containing fragments of huntingtin in spiny neurons	Striatum	[125, 153–155]
Amyotrophic lateral sclerosis	Affection of motor neurons caused by compromised production of superoxide dismutase	Cerebral cortex, spinal cord	[156–160]
Wilson’s disease	Mutation in the gene encoding a copper-transporting protein ATP7B	Striatum	[161–165]
PKAN	Autosomal recessive disease involving multiple genes characterized by excessive iron accumulation in the CNS	Striatum	[166–170]

*CNS* central nervous system, *PKAN* pantothenate kinase-associated neurodegeneration

Neurodegenerative disorders are also adjoined by various degrees of aseptic inflammation and iron accumulation [2–5]. Inflammatory cells are often present in the vicinity of the affected neurons with varying appearance ranging from robust in Alzheimer’s disease to somewhat slighter in Parkinson’s disease and amyotrophic lateral sclerosis (ALS). The inflammatory process accompanying the degenerating involves glial cells of the CNS, mainly astrocytes and microglia, and monocytes of the circulation that migrate into the CNS to transform into phagocytic macrophages [6–11]. The presence of phagocytic monocytes migrating into the CNS together with local recruitment of activated microglia denotes a prominent apparatus for killing and phagocytosis of damaged and dying neurons [12].

**Fig. 1:**
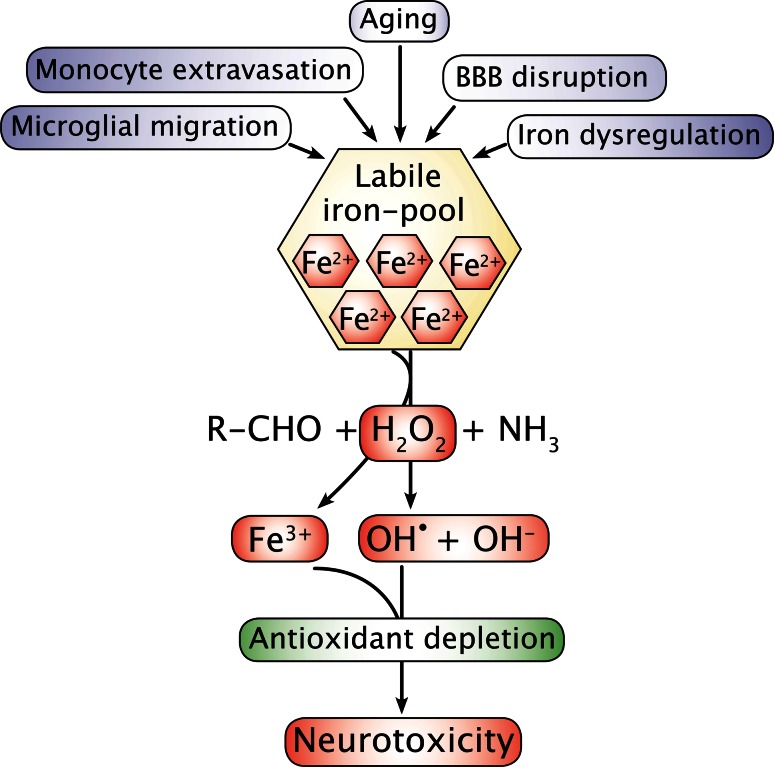
Overview of major events that lead to iron accumulation in the central nervous system. Dysregulation of cellular iron homeostasis is likely to happen if ferritin expression is hampered leading to a failure in the binding of residual iron. Iron may also pathologically accumulate inside cells due to inhibition of ferroportin mediated by hepcidin (see also text and Fig. 5). Iron accumulating in cells is likely to play a significant role for initiating neurodegeneration via promoting free radical formation by Fenton chemistry. Ferrous iron (Fe^2+^) of the labile iron pool gets catalyzed to ferric iron (Fe^3+^) in a chemical reaction mediated by hydrogen peroxide (H_2_O_2_) formed as a byproduct of oxidative metabolism, which leads to formation of hydroxide radical (OH^•^). Depletion in antioxidants inside the cell may fail to scavenge hydroxide radicals and propagate neurotoxicity. *BBB* blood–brain barrier

Characteristically, all disorders mentioned in Table 1 also lead to iron accumulation in the areas affected by neurodegeneration; a feature different from that of the aging CNS, which also increasingly accumulates iron but without adjoining inflammation [13–17]. Iron-containing inflammatory cells, including microglia and macrophages, are consistently present in inflamed brain tissue, suggesting the latter as sources for both iron donation and contribution to production of reactive oxygen species (ROS) via release of free radicals as part of their respiratory burst activity [18, 19] (Fig. 1).

### Outline

The changes in the concentration of transient metals like iron, copper, and zinc with increasing age are general phenomena with the increase in iron being the most notable [3, 15]. The sources explaining the additional increase in iron in neurodegeneration are reasonably mainly external. Migration of inflammatory cells from the periphery thus may pave the way for the iron accumulation known to take place in the degenerating CNS, as monocytes that transform into tissue macrophages while migrating passed the blood–brain barrier into the CNS contain a high concentration of labile iron and the iron-storing protein ferritin capable of binding approximately 4,500 atoms of iron to each ferritin molecule [18, 20]. We hypothesize that macrophages, which participate in the phagocytosis of damaged and dying cells, are likely to die out themselves, leading to the release of their iron content inside the CNS. The iron could transform from the repository of ferric iron present inside ferritin to the more available but also labile ferrous iron that might contribute to production of ROS.

Apart from the interests in the contribution of iron for ROS production, little activity has been devoted to the mechanisms underlying the causes of deposition of iron in CNS areas affected with neurodegeneration. The main topic of this review is therefore to cover the significance of the iron carried into the CNS by circulatory monocytes during the process of inflammation, its contribution to neurodegeneration, which clearly occurs in mechanisms different from the handling of the increasing levels of iron in the CNS during normal aging and the therapeutic potential of preventing migration of iron-containing phagocytic macrophages into the CNS.

## Ferritin concomitantly increases in the normal aging brain to scavenge excess iron

Throughout life, the CNS continuously takes up iron from the circulation by means of receptor-mediated endocytosis of iron-transferrin by brain capillary endothelial cells denoting the blood–brain barrier [21]. The CNS does not excrete iron to the same extent, explaining why the CNS’s turnover of iron is extremely low [16, 22]. Reflecting the increasing iron concentration of the aging CNS, iron distributes to all cell types but its detection is hampered by that only iron on its oxidated ferric form can be detectable using histological approaches. Neuronal iron almost exclusively appears in neurons on its ferrous form [23] and does not appear in neurons as ferric iron until aging [23–25]. The cells of the normal aging CNS generally seem capable of handling the increasing iron as they readily respond by increasing their content of ferritin [26]. At the cellular level, the main feature of the aging CNS is that oligodendrocytes substantially increase their ferritin protein expression, which is a dramatic change that represents a raise from virtually no expression during development through an intermediate in the normal adult CNS [27]. Neurons of many brain regions also increase their iron and ferritin content with increasing age, but in a much more heterogeneous pattern than seen in oligodendrocytes, suggesting that the handling of iron by neurons differs among various regions of the CNS. Microglia also increasingly expresses ferritin with aging, whereas astrocytes paradoxically maintain a virtual complete lack of ferritin protein in the development CNS and throughout adulthood and aging despite the fact that they have the capacity to take up iron [28].

Mechanistically, being part of the Fenton and Haber–Weiss reactions iron can contribute to neuronal oxidative stress and damage by participating as ferrous iron, i.e., on its reduced form) (Fig. 1). However, as the increasing concentration of iron is reflected by a parallel increase in ferritin in the aged CNS, iron is likely to occur on its oxidized ferric form due to the ferroxidase activity denoted by ferritin [29]. Hence, the ready translation of ferritin mRNA in response to increasing iron levels as well as the enormous capability to bind iron makes it likely that sufficient ferritin expression is proper relative to the cellular level of iron during aging, which thus prevents ferrous iron from participating in unwanted ROS formation [26, 29].

Concomitant to the increasing content of iron of the aging CNS, ROS formation also increases, which is attributed to a lower functioning of the enzymes of the mitochondrial respiratory chain in addition to a weakened antioxidative defense from molecules such as glutathione [30, 31]. Hence, while important to scavenge the risks of the increasing iron for catalyzing free radicals, ferritin is simultaneously at risk of being damaged by ROS itself, which could release iron from ferritin and subsequently allow unbound iron to enter the pool of labile iron known to be chemically much more reactive than ferritin-bound iron [29, 32]. The increasing ROS formation could also hamper the functioning of iron-responsive proteins or their transcription, which may further impede the regulation of ferritin mRNA translation [33]. The risk is therefore that the increased ROS formation in the aging CNS, incompletely compensated by an antioxidant response, damages ferritin with a resulting release of iron and leading to a further increase in ROS production, hence creating a vicious cycle [29, 34]. Human cases and animal models mutated in ferritin achieve a neurodegenerative state supporting that mismatch in the capability to increase the expression of ferritin in response to increasing iron leads to neuropathology [34, 35].

## Oxidative stress formation in the inflamed CNS receives a significant contribution from iron-containing macrophages

During the processes of oxygen metabolism and ATP formation, mitochondria form ROS and reactive nitrogen species (RNS) as by-products [30, 36, 37], and oxidative stress occurs when the formation of ROS and RNS exceeds the elimination capacity of antioxidative defense system [38, 39]. The CNS receives approximately 20 % of the blood supply of the entire body, and the extraction of oxygen from the blood by the CNS is concomitantly high, making the microenvironment of the CNS rich in oxygen radicals [40]. The tissue of the CNS is rich in peroxidized fatty acids, and even though the cells of the CNS also harbor an antioxidant defense system, the CNS is highly prone for impact by ROS and RNS [41]. Oxidative stress generally plays a role in disease pathogenesis in consequence of distressed metabolism in the CNS because of the toxicity of ROS produced in neurons and inflammatory cells. Many cellular biochemical reactions are responsible for the production of ROS and also RNS in the presence of nitric oxide (NO). The ROS are free radicals of great physiological importance for cells of the innate immune system like monocytes and macrophages functioning to eliminate invading pathogens and dying cells following phagocytosis, while migrating into the CNS in various disease conditions [2, 4–8]. However, the ROS released from monocytes and macrophages are also potentially harmful to tissues of the CNS, because of their ability to react with almost any cellular component including DNA, lipids, and proteins [42].

The ROS-producing macrophages entering the brain can be functionally sub-categorized into two distinct groups; the pro-inflammatory M1 phenotype and the anti-inflammatory M2 phenotype [43, 44]. Different iron handling between these subgroups of macrophages can be observed in various conditions with chronic inflammation outside the CNS. While activated M1 macrophages readily increase their iron-content, e.g., via DMT1, M2 macrophages are characterized by a much lower iron content due to continuous release of iron through the iron-exporter ferroportin [44, 45]. Being resident macrophages, microglia can also be subdivided into M1 and M2, both of which have been described in areas of neurodegeneration [8]. The participation from each of these subtypes in chronic neurodegenerative disorders are generally less accounted for compared to the macrophages of peripheral tissues, and therefore cannot be taken into consideration on the distribution and functionality of macrophages entering the brain [8]. However, evidence from transgenic Alzheimer’s disease mice suggests that a phenotypic shift from M2 to M1 microglia occurs in models of prolonged neuroinflammation potentially aggravating neuronal degeneration with increasing age [46]. Furthermore, inflammatory stimulation of microglia leads to expression of DMT1 (normally undetectable in resting microglia), which possibly accounts for the increased iron content of activated microglia in regions affected by neurodegeneration [47].

ROS are not only produced by mitochondria but also by nicotinamide adenine dinucleotide phosphate (NADPH) oxidase, an enzyme found in the plasma membrane of all cell types of the CNS, which forms superoxide when metabolizing molecular oxygen [48]. There is strong evidence that NADPH oxidase is upregulated in affected regions during neurodegeneration, e.g., in an experimental model of Parkinson’s disease, activation of macrophages and microglia induced by NADPH oxidase triggered a subsequent production of ROS, thus damaging adjacent neurons, suggesting that the inflammatory process affecting a single region may spread to adjacent regions and hamper otherwise unaffected/healthy neurons [49–51]. Furthermore, depletion of the peripheral functioning monocytes may lead to improved neural outcome, indicating that migration of monocytes into the CNS is of significance [52, 53].

**Fig. 2:**
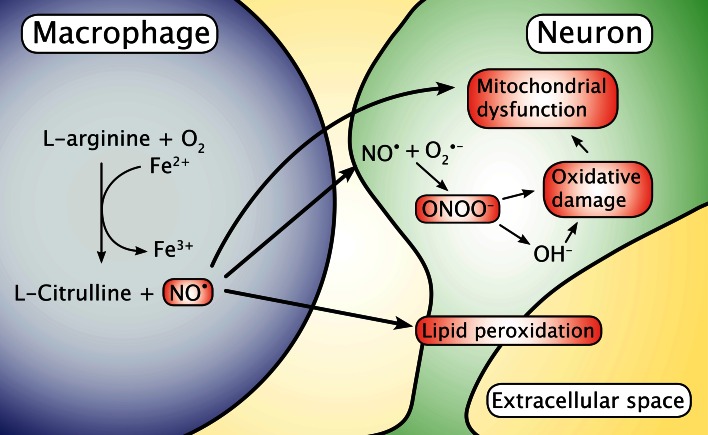
Macrophages migrating into the brain release nitric oxide radicals (NO^•^), a process that involves the catalytic oxidation of ferrous iron. NO^•^ is capable of diffusing pass the cellular membranes and into neurons where it can react with superoxide (O2^•−^) and promote formation of the highly reactive and toxic peroxynitrite (ONOO^−^)

Another possible contributor to the oxidative environment in neurodegenerative disorders is NO released from CNS macrophages. The NO can diffuse into neurons from the vicinity of the macrophages and catalyze the formation of the damaging pro-oxidant peroxynitrite via chemical reaction with neuronal superoxide (Fig. 2) [54–59]. NO is also known to interact directly on the binding of iron to ferritin, as NO can release iron from ferritin, causing iron to appear on reactive loosely bound forms [60].

**Fig. 3:**
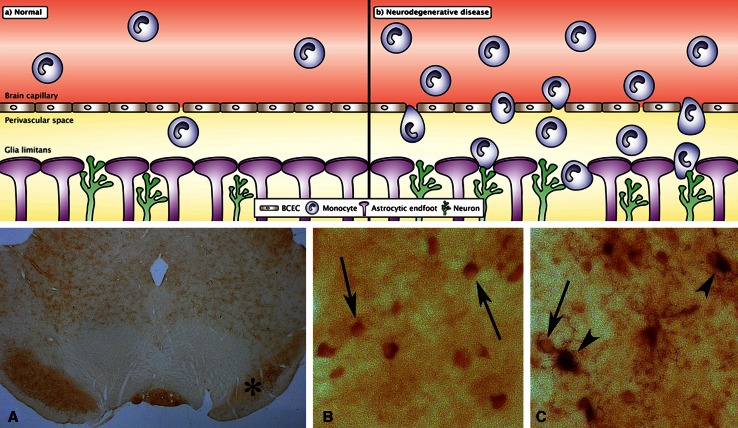
Migration of monocyte into the normal (**a**) and neurodegenerating (**b**) brain. The monocytes migrate through the brain capillaries that form the blood–brain barrier even in normal conditions. In conditions with pathology, the migration of monocytes into the brain through the blood–brain barrier is much more pronounced. In consequence of this migratory process, the monocytes transform into macrophages with immediate access to neuronal projections and astrocytic end-feet forming the so-called glia limitans, demarcating the brain perivascular space. *BCEC* brain capillary endothelial cells. *Bottom* Inflammatory cells in the substantia nigra reticulata identified by ferritin labeling. Rats were injected unilaterally into the striatum with a glutamate agonist to induce degeneration in the striatal nigral pathway. The loss of striatal innervation of the substantia nigra reticulata leads to a gradual degeneration that is followed by chronic inflammation and iron accumulation (Sastry and Arendash, 1995). **A** The mesencephalon with the affected substantia nigra reticulata indicated (*asterisk*). **B** In the substantia nigra reticulata of the unaffected side, the ferritin-containing cells are virtually only seen in oligodendrocytes (*arrows*), whereas in the affected substantia nigra reticulate (**C**), ferritin-containing cells are identified as monocytes and macrophages (*arrowheads*) and oligodendrocytes (*arrow*) (Thomsen and Moos, in preparation). Magnification: **A** ×5. **B**–**C** ×200

The migration of monocytes into the CNS is dramatically increased in virtually any condition with pathology inside the CNS (Fig. 3) [6–11]. Concomitantly to the increase in this migration, the concentration of iron increases in affected CNS regions, e.g., the concentration of iron increases in the substantia nigra and striatum in Parkinson’s disease, in hippocampal and many forebrain regions in Alzheimer’s disease, and in the striatum in Huntington’s disease [61–63], which makes it obvious to suggest that the migration of iron-containing monocytes into the diseased CNS explains the increasing concentration of iron, which will be dealt with in the following section.

## Iron-mediated pathology in neurodegeneration as interplay between increased deposition, oxidative stress formation, and impaired iron efflux

In accordance with our hypothesis, accumulation of iron in the CNS during neurodegenerative diseases could be attributable to inflammatory cells migrating into the affected areas and deposit iron [5]. Other possible sources for chronic iron deposition are due to changes in the physiological transport of iron across the blood–brain barrier and an impaired cellular capability to export iron. The following paragraphs are devoted to these issues via a sequential coverage of: (i) The blood–brain barrier in inflammatory conditions; (ii) accumulation of inflammatory cells and iron deposition in both non-neuronal and neuronal tissues; (iii) transport of non-cellular iron across the blood–brain barrier in chronic CNS inflammation, and (iv) compromise in the capability to export iron from the CNS.

### The blood–brain barrier in inflammatory conditions

The blood–brain barrier, consisting of non-fenestrated capillary endothelial cells with their intermingling tight junctions, denotes a morphological interface situated between the circulation and the CNS parenchyma [64, 65]. These endothelial cells prevent large, especially lipid insoluble, molecules from entering the CNS and also closely regulates the level of nutrients, vitamins, and minerals that enters the CNS. An extended interface between the blood and the CNS is formed by the brain capillary endothelial cells, their basement membrane, and glial cells such as astrocytes and pericytes, which together form the so-called neurovascular unit [64]. While not directly involved in regulating barrier permeability, these glial cell types are important for the induction and maintenance of the barrier characteristics of the brain capillary endothelial cells [66].

The integrity of the blood–brain barrier is reportedly compromised and thought to play a significant role in many different neurological disorders, including epilepsy, migraine, stroke, and various neurodegenerative disorders [67–70]. Among the latter, disruption of the blood–brain barrier was described in Alzheimer’s disease, Parkinson’s disease, and amyotrophic lateral sclerosis [71–74]. In Alzheimer’s disease, inflammation of the cerebral vasculature seems to be an early event in the progression of neuroinflammation and Aβ deposition [75, 76]. Early inflammation of the blood–brain barrier was recently observed together with changes in permeability and upregulation of MECA-32 and selectin expression in experimental models of neurodegeneration [67, 68, 70]. Hence, activation of the endothelium coincides with early leakage of the blood–brain barrier, which may allow for inflammatory cells like monocytes and macrophage to enter the CNS locally early in disease to initiate deposition of iron. Inflammation of the periphery may also lead to opening of the blood–brain barrier, increased migration of monocytes into the CNS, microglial activation, damage to dopaminergic neurons, and exaggerated deposition of iron inside the CNS [47, 52, 53].

### Deposition of iron in non-neurological disorders is associated with ROS formation and chronic inflammatory pathology

Migration of monocytes through an endothelial barrier is characteristic for several chronic inflammatory conditions in non-neuronal tissues. The monocytes harbor the largest pool of labile iron among the hematopoietic cells [18]. This labile iron pool is important for the adhesion of the monocytes to endothelial cells, and subsequently their migration through endothelium in non-neuronal tissues and their transformation into macrophages [77, 78]. This labile iron pool is, however, loosely bound to proteins and prone to participate in cellular destructive reactions when monocytes are attracted to regions of tissue inflammation [18]. Inflammation and essential metals are highly entwined with respect to diverse peripheral chronic inflammatory disorders. The role of iron as a potential catalyst of inflammation and degeneration in non-neuronal tissues has been investigated intensely with regards to the chronic inflammation that occurs in various conditions such as joint diseases, atherosclerosis, and inflammatory bowel disorders. The literature discloses evidence that iron deposits in synovial fluid in numerous inflammatory and degenerative joint disorders, e.g. rheumatoid arthritis, osteoarthritis, hemophilic synovitis, and seronegative arthritis [79–86]. In these joint diseases, just as in inflammatory disorders of the bowel, accumulating iron seemingly takes on a part as a villain to maintain and exacerbate chronic inflammation.

Perhaps the most obvious example of how monocytes and macrophages may deposit iron and exacerbate pathology is seen in atherosclerosis, which shares similarities with the inflammation of the CNS with respect to the presence of endothelial abnormalities, transmigration of monocytes through activated endothelial cells, local recruitment of macrophages to the sites of inflammation, and accumulation of iron [87]. In atherosclerosis, the process of chronic vascular inflammation gets significant contribution from circulating iron-containing monocytes that migrate into the sub-endothelial compartment attracted primarily by the endothelial expression of adhesion molecules like vascular cell adhesion molecule-1 (VCAM-1), intercellular adhesion molecule-1 (ICAM-1), and chemo-attractant chemokine (C–C motif) ligand 2 (CCL2), molecules also being expressed by the activated brain endothelium in inflammatory conditions in the CNS [88–90]. In the inflamed subintimal zone of the arteries, the labile iron present in macrophages is likely to play a detrimental role as it prompts formation of ROS and free radicals through the Fenton and Haber–Weiss reactions [91, 92]. Accumulation of iron in non-neurological diseases, particularly atherosclerosis, thus supports the notion that inflammatory cells migrate into inflamed tissues where they deposit iron and thereby contribute to ongoing inflammation.

### The accumulation of inflammatory cells and iron deposition in neuronal tissue

Monocytes migrate through the blood–brain barrier in brain ischemia, transform into tissue macrophages, and carry iron into the brain where it exerts deleterious effects [93, 94]. The migrating monocytes therefore denote a plausible source of iron for the affected areas in neurodegenerative disorders (Fig. 3). Similar to the events taking place during inflammation in non-neuronal tissues, in inflammatory conditions of the CNS, the expression of selectins by brain capillary endothelial cells leads to attachment of monocytes that “roll” in the direction of the circulation towards the endothelial surface. After adhesion to immobilized chemokines, the integrins of the migrating monocytes bind to ligands, e.g., ICAM-1, which in turn leads to a tighter adhesion of the monocytes to the surface of brain capillary endothelial cells. Subsequently, protruding processes of the monocytes are likely to facilitate the search for chemokines expressed on the abluminal surface of the brain capillary endothelial cells, which enable their further entry into the perivascular space when passing though the brain endothelium. Here, the monocytes may additionally secrete matrix metalloproteinases to degrade the extracellular matrix of the basement membrane or bind to the extracellular matrix through β1-integrin, thereby facilitating the final migration into the brain parenchyma [95]. The presence of Aβ, either on its soluble form near the luminal side of brain capillary endothelial cells or on its aggregated form on their abluminal side, significantly potentiates the transmigration of monocytes in an in vitro model of the blood–brain barrier [96] and clearly suggests that monocytes of the periphery enter the CNS in pathological conditions.

**Fig. 4:**
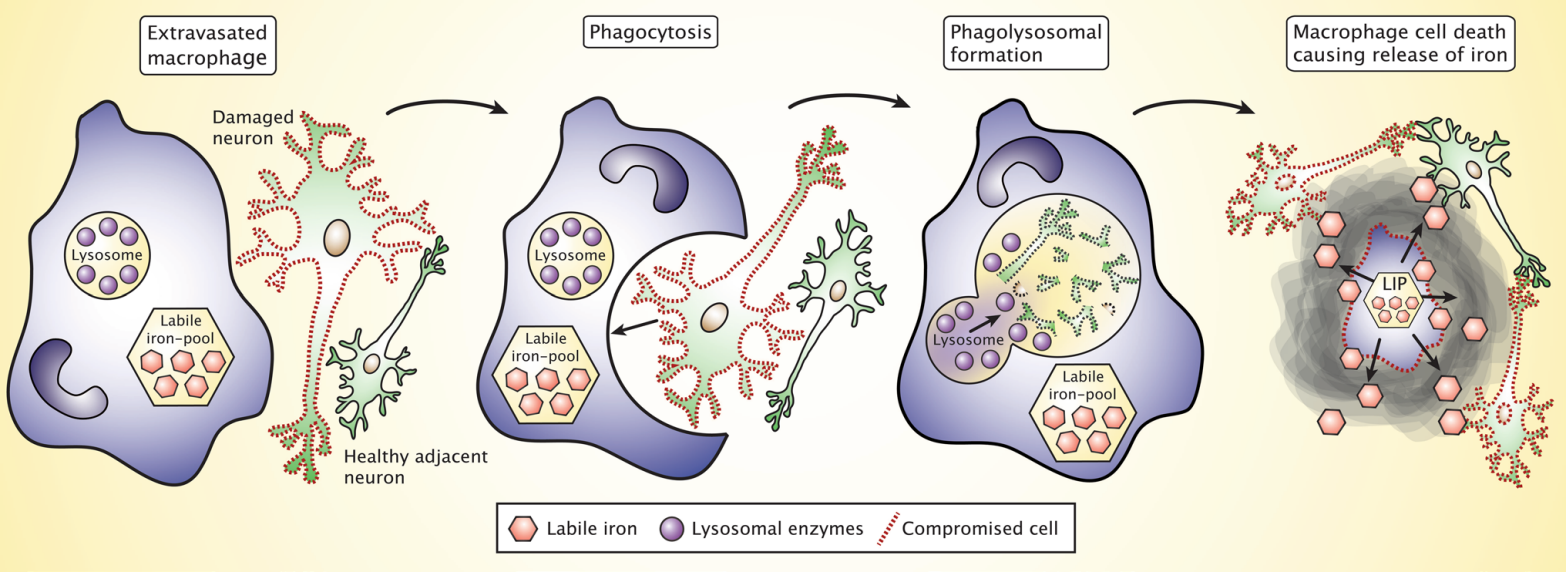
Extravasated macrophages phagocytose and degrade damaged neurons and subsequently die to terminate their function, which leads to the release of iron into the extracellular space of the CNS on a low molecular weight form

There is strong evidence from experimental studies supporting that pathological conditions of the CNS leads to iron accumulation in affected brain regions, e.g., chemical lesion of the striatum leads to degeneration accompanied by manifest inflammation and iron deposition not only in the striatum but also significantly in the substantia nigra that communicate bilaterally with the striatum [5]. Hence, in good agreement with our hypothesis, macrophages migrating into the substantia nigra may phagocytose damaged neurons and subsequently deposit iron. Therefore, the chronic migration of monocytes could also play an important role in the progression of neurodegenerative disorders (Fig. 4) [60]. While acting as activated phagocytic cells, the macrophages release NO that can directly promote iron release from ferritin [59]. Moreover, the macrophages will eventually die out by apoptosis to terminate the inflammatory process unless chronic stimuli precedes [59, 97, 98]. This will lead to release of the labile iron present inside the macrophages, which subsequently becomes accessible for both already damaged and otherwise healthy neurons (Fig. 4). The iron released from dying macrophages can play a significant role not only for direct neuronal damage and resulting cell death but also by means of ROS-mediated post-translational affection of proteins to gradually perturb their function, e.g., by promoting neuronal fibril and aggregate formation as seen in Parkinson’s disease that eventually could also lead to neuronal cell death, but in a much more long-lasting scenery [99, 100].

In the resting brain, entering macrophages can differentiate into microglia [6, 11, 101]. Activation of microglia is a well-described phenomenon occurring in a number of neurodegenerative disorders due to molecular release from dying neurons of ROS, chemokines, interleukins, and tumor necrosis factor α (TNF-α) [8, 102] that leads to the migration of the microglia towards the regions affected of neurodegeneration, where they participate in the phagocytosis and destruction of dying neurons until they die out themselves by apoptosis [12, 97, 103–105]. Microglia contain relatively high amounts of iron bound to ferritin and the migration of microglia to the degenerating regions therefore make a significant contribution to the local increase in iron by a mechanism similar to the mechanism proposed to occur in macrophages [106]. Moreover, the activation of microglia is known to facilitate the release of labile iron from the ferritin complex, hereby introducing free iron to neurons in the areas of disease [107].

### The transport of non-cellular iron across the blood–brain barrier in chronic inflammation of the CNS

The regulation of iron uptake in the brain is a tightly controlled process involving binding of transferrin to the brain capillary endothelial cells followed by endosomal uptake of holo-transferrin [108]. On the abluminal surface of the brain capillary endothelial cells, the microenvironment of the CNS could drive the release of iron, which would subsequently be bound to parenchymal transferrin, citrate or ATP, and passes into the brain interstitium for uptake by neuronal and glial uptake. Inflammatory activity within affected regions of the CNS could lead to iron being released from macrophages that could bind to transferrin in the extracellular space and enter neurons by means of receptor-mediated uptake of iron transferrin or by non-transferrin bound iron uptake, which would also apply to glial cells [109].

The fact that increasing the concentration of iron in plasma still fails to improve the entry of iron into the CNS both experimentally or in human conditions with hemochromatosis indicates that the brain capillary endothelial cells with intact blood–brain barrier properties are able to down-regulate their expression of transferrin receptors in response to the increasing availability [108]. By contrast, in conditions with neurodegeneration and inflammation of the CNS, there is evidence for an increased opening of the blood–brain barrier leading to paravascular passage of macromolecules locally in regions with affected neurons [68, 110].

The activation of the endothelium in Alzheimer’s disease with down-regulation of tight junction expression precedes the deposition of Aβ but coincides with early blood–brain barrier leakage, which could represent the earliest stages of disease [75, 76]. This raises the question if paraendothelial transfer of holo-transferrin through these leakages may contribute to the increased deposition of iron in many neurological disorders. An increase in iron entry through a compromised blood–brain barrier with increased paraendothelial transfer was recently reported in an experimental model of transient forebrain ischemia [93, 94], but quantitative evidence that could justify whether iron-transferrin entering the brain due to a compromised blood–brain barrier to yield significances in iron accumulation is still needed.

### Compromise in the capability to export iron from the CNS

In addition to its participation in intestinal absorption and circulatory iron homeostasis via expression in duodenal enterocytes, hepatocytes, and macrophages, ferroportin is also expressed in neurons of the CNS with significant regional variations [111–113]. Ferroportin is the only described protein known to mediate cellular efflux of iron [114], which would make a mismatch in the functionality of ferroportin in the CNS a possibility for iron to get trapped inside neurons, leading to their lack of capability to excrete iron. In turn, this incapability to export iron from cells would lead to accumulation of iron inside CNS and pose neurons to an increased risk for ROS-mediated damage.

Ferroportin expression is post-transcriptionally regulated via interaction between iron-regulatory proteins and an iron-responsive element present in the 3′-end of its mRNA, indicating that ample presence of iron leads to stabilization and increased half-life of ferroportin mRNA leading to higher translation [114]. The expression of ferroportin is also post-translationally regulated by the hormone hepcidin, as the binding of hepcidin results in phosphorylation, internalization, and degradation of ferroportin, thus severely altering the ability of the cell to excrete iron [115, 116].

**Fig. 5:**
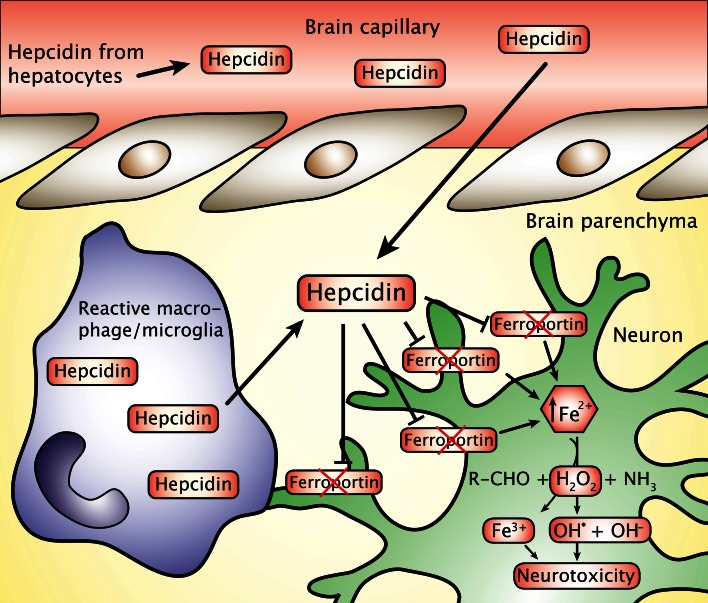
The macrophages, like monocytes and microglia, are capable of secreting hepcidin into the brain extracellular space. Hepatic hepcidin is synthesized in response to inflammatory signals and secreted into blood plasma from where it can diffuse into the brain in areas with a compromised blood–brain barrier. The hepcidin is capable of binding and inhibiting ferroportin needed for export of iron from neurons, which may result in neuronal iron accumulation and increased the likelihood of neuronal damage via Fenton chemistry

Experimental neurodegeneration has never been correlated directly with changes in neuronal ferroportin expression, but injections of hepcidin into the lateral ventricle of the rat leads to a decrease in neuronal ferroportin, confirming the notion of a degradation of ferroportin in the presence of hepcidin [117]. Hepcidin is secreted from hepatocytes in systemic inflammatory conditions, and provided that pathological activity of the CNS leads to humoral signaling in the circulation, it is predictable that such activity would lead to raise hepcidin in plasma with the likelihood of passing into the brain, provided the integrity of the blood–brain barrier gets compromised. Clearly, a condition with chronic pathology of the CNS leading to migration of iron-containing macrophages and their subsequent demise as discussed in previous paragraphs would make it likely that the neurons could suffer from the accumulation of iron released from dying macrophages combined with the incapability to release iron via ferroportin due to the presence of hepcidin inside the inflamed CNS (Fig. 5). This notion gets exaggerated by the fact that the turnover of iron in the CNS is very slow even in the normal brain, which can be attributed to iron being accumulated in cells of the brain rather than being transported out of the brain (cf. [118]).

## Pharmacological intervention to limit pathological iron deposition in neurodegenerative disorders

The chronic setting of iron-containing macrophages migrating into the CNS in affected areas in conditions with neurodegeneration gives rise to deposition of iron that eventual may reach limits that will promote formation of free radicals to an extent uncontrollable by increases in ferritin expression. Therefore, it is reasonable to consider the possibilities of pharmacological intervention to limit the damages of pathological iron deposition. A first strategy will be to directly intervene into the events leading to neuronal degeneration, but the coverage of this option goes beyond this review. Further downstream is the cause of inhibiting migration of monocytes into the brain, followed by antioxidant therapy to neutralize the formation of excess free radicals, and final the possibilities of limiting iron deposition by means of chelator therapy that would serve to reduce iron occurring extracellularly due to degradation of damaged and dying cells including brain macrophages and intracellularly in neurons.

### Inhibition of macrophages in neurodegeneration using antioxidant therapy

The local environment in affected areas in neurodegenerative disorders is enriched in oxidants and the load from free radicals is permanently at risk for exaggerating neuronal cell death. The contribution of pro-oxidants can in part be attributed to the action of monocytes migrating through the blood–brain barrier and microglia migrating inside the CNS towards the sites of neurodegeneration. Hence, the inhibition of migration of monocytes into the CNS could be of significance to halt disease progression and contribute to the development of effective therapeutic regimens in conditions with neurodegeneration [119]. The action of migrating macrophages can qualitatively be inhibited by treatment with antioxidants that serve to reduce the load of pro-oxidant molecules, although clinical significance of antioxidant treatment in neurodegenerative diseases is yet to be proven. The impact of corticosteroids and several non-steroid anti-inflammatory drugs on the progression of Alzheimer’s disease was tested in a clinical trial, but beneficial effects could not be detected [120, 121]. Among other promising and diverse agents such as co-enzyme Q-10 (mitochondrial enhancer and antioxidant), minocycline (anti-inflammatory agent), and rasagiline (MAO-inhibitor), none has yet made it to the clinic [122]. Minocycline is highly lipophilic and hence readily crosses the blood–brain barrier [123]. This advantage in the pharmacokinetic profile would suggest the compound to be preferable for treatment of chronic neurodegenerative disorders, and irrespective of the mechanisms that could be beneficial in the usage of this anti-inflammatory drug, its success in clinical testing will probably depend on the length of the dosage regimen and possibly also that the onset of therapy takes place when the disease is not too progressed. Although pivotal progress has not been made, several compounds affecting oxidant levels were suggested for further exploration for the treatment of Parkinson’s disease and research into these compounds and their promising derivatives continues [122, 124]. This also applies to treatment of Huntington’s disease, where efforts focus on increasing autophagy and ubiquitination of mutant huntingtin protein mHtt combined with treatment of antioxidants like co-enzyme Q10 and cysteamine [125, 126].

### Iron chelators for neutralizing the effects of iron accumulation in neurodegeneration

Iron chelating agents were examined in various degenerative disorders with iron pathogenically involved, e.g., in Parkinson’s disease, coronary heart disease, and general atherosclerosis. Studies in rodents suggest that restriction in iron intake reduces progression in atherosclerotic plaque formation [77, 127, 128], while administration of excess iron on the contrary augments these processes [129]. Furthermore, treatment with the iron chelating agent desferrioxamine has proven successful in reducing iron concentration in atherosclerotic plaques and decreasing atherosclerotic lesion formation in animal models [130, 131]. In neurodegeneration, a principal target for iron chelation is extracellular iron due to degradation of damaged and dying cells including brain macrophages. As stated in the preceding paragraphs, the macrophages entering the CNS are likely to die out by apoptosis when having commenced their function, which will lead to their release of iron into the extracellular space. The iron released in such a condition will likely bind to transferrin or low molecular weight substances like citrate and ATP [118] and be amendable for therapy with extracellularly acting iron chelators, provided these are capable of passing the blood–brain barrier. Conversely, iron accumulating in neurons as part of the general aging and iron taken up in excess from the extracellular space due to the degeneration of other neurons and macrophages will need the action of intracellular iron chelators.

In spite of iron chelators like desferrioxamine and deferiprone having been used beneficially for decades for the treatment of hemochromatosis and iron poisoning, the usage of iron chelators in neurodegenerative disorders has not resulted in pivotal clinical breakthroughs [132]. Desferrioxamine and deferiprone used clinically to treat thalassemia via parenteral injection were shown to successfully reduce striatal DA neuron depletion and behavioral symptoms in the 6-hydroxydopamine (6-OHDA) model of Parkinson’s disease after injection into the cerebral ventricles [133, 134]. As desferrioxamine does not penetrate the blood–brain barrier, this drug has obvious limitations for treatment inside the CNS, but it might be valuable for chelation of iron extracellularly in the brain, in particular in pathological conditions with a perturbed blood–brain barrier. This condition does not fully apply to deferiprone that crosses the intact blood–brain barrier to some extent [135]. Interestingly, deferiprone is currently being examined in clinical studies for its capability to reduce disease progression in PKAN [136, 137].

**Fig. 6:**
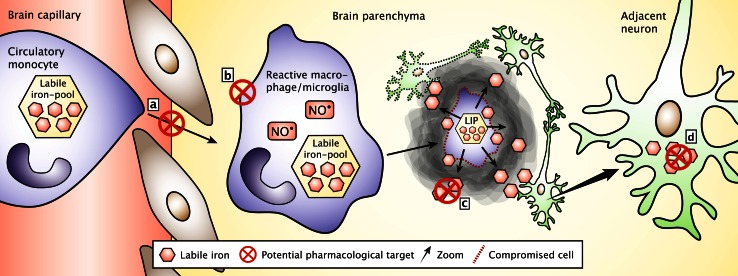
Potential pharmacological intervention points to inhibit the impact of migrating macrophages on their deposition of iron in the brain. **a** Inhibition of monocytes migration into the brain via transfer through the brain capillaries. **b** Inhibition of the functioning of the brain macrophages for phagocytosis and nitric oxide (NO^•^) release. **c** Extracellular chelation of low molecular weight iron released from dying macrophages. **d** Intracellular chelation of iron in neurons subsequent to their uptake of low molecular weight iron from the extracellular space

An alternate application of oral iron chelators is to use the oral route, which is particularly relevant for lipophilic drugs. The lipophilic molecule deferasirox was also shown to attenuate the loss of dopaminergic neurons and preserve striatal dopamine in the 6-OHDA model of Parkinson’s disease [134]. Deferasirox has been approved by the FDA for therapy in conditions with chronic iron overload. Contrary to the parenteral iron chelators, deferasirox may exert its action inside neurons rather than in the extracellular environment. This indicates that it could be favorable to combine different iron chelators for studies in experimental models with iron accumulation in an attempt to simultaneously chelate iron deposited extracellularly occurring from cellular debris of dying neurons and macrophages and iron accumulating inside neurons due to uptake from the pathologically iron-enriched immediate interstitium. The strategy of combining iron chelators acting on iron present extra- or intracellularly might even benefit from a combination with therapeutics to inhibit migration of iron-containing macrophages into the CNS (Fig. 6).

## Conclusions

For reasons still unknown, the concentration of iron increases in the brain with increasing age. The rise in brain iron content in aging is even higher in neurodegenerative disorders, which is probably a result of the inflammatory process occurring in areas affected by neurodegeneration. The transformation of iron-containing monocytes that migrate across the blood–brain barrier into the CNS while transforming into brain macrophages is in agreement with this notion. As a consequence of the phagocytosis of damaged and dying neurons, the entering macrophages eventually die out, leading to the release of their own content of iron into the brain interstitium from where it will enter the pool of loosely bound iron that catalytically may promote formation of free-radical-mediated stress and oxidative damage to cell membranes in the adjacent environment. Damaged but also healthy neurons may engulf iron of the dying macrophages from extracellular space leading to cellular accumulation with the risk of further promoting neuronal damage. The chronic inflammation and decreased blood–brain barrier integrity accompanying neurodegenerative disorders may be pharmacologically managed via intervention of the monocyte migration into the brain combined with chelator therapeutics that aim to chelate iron released extracellularly due to release from dying macrophages and chelating of iron deposited intracellularly in neurons.

## Abbreviations

CNS: Central nervous system
DMT1: Divalent metal transporter 1
IL: Interleukin
PKAN: Pantothenate kinase-associated neurodegeneration
NADPH: Nicotinamide adenine dinucleotide phosphate
NO: Nitric oxide
RNS: Reactive nitrogen species
ROS: Reactive oxygen species
TNF-α: Tumor necrosis factor α
6-OHDA: 6-hydroxydopamine
